# Discovery and Comprehensive Characterization of *Pseudomonas* sp. MUP55: Taxonomy, Massetolide-Mediated Biocontrol, and Regulatory and Antimicrobial Contributions of the *pvf* Cluster

**DOI:** 10.3390/ijms27156749

**Published:** 2026-07-28

**Authors:** Hussain Alattas, Samuele Sala, Joseph Boctor, Crystal E. Young, Daniel V. Murphy, Colin Scott

**Affiliations:** 1 Bioplastics Innovation Hub, Food Futures Institute, Murdoch University, Murdoch, WA 6150, Australia; joseph.boctor@murdoch.edu.au (J.B.); c.young@murdoch.edu.au (C.E.Y.); daniel.murphy@murdoch.edu.au (D.V.M.); colin.scott@murdoch.edu.au (C.S.); 2 The Australian National Phenome Centre and Computational and Systems Medicine, Health Futures Institute, Murdoch University, Harry Perkins Building, Perth, WA 6150, Australia; samuele.sala@murdoch.edu.au

**Keywords:** secondary metabolites, biocontrol, cyclic lipopeptides

## Abstract

*Pseudomonas* sp. MUP55, isolated from rainfall water in Western Australia, was characterized by polyphasic taxonomy and functional assays. Whole-genome and 16S rRNA phylogeny placed *Pseudomonas* sp. MUP55 in the *Pseudomonas fluorescens* species group. Massetolide A/D was identified as the leading candidate bioactive compound(s), consistent with its biosynthetic gene cluster, GNPS library matching, and loss of activity in regulatory mutants. The strain showed broad-spectrum antimicrobial activity against bacterial (*Escherichia coli* and *Xanthomonas campestris*) and fungal (*Fusarium oxysporum* and *Rhizoctonia solani*) plant pathogens. GacA regulates Massetolide production: a P58L mutation abolished synthesis and reduced biocontrol efficacy. Metabolomic and transcriptomic analysis of a Δ*pvfC* mutant revealed that the *pvf* cluster regulates specialized metabolism while also contributing to secreted growth-inhibitory activity. The *pvf* cluster differentially regulates dual siderophore systems and uncouples the co-regulated small RNAs rsmY and rsmZ in the Gac/Rsm cascade. Deletion of *pvfC* partially reduced the growth-inhibitory activity of *Pseudomonas* sp. MUP55 supernatants against bacterial pathogens, indicating that *pvfC* also influences secreted antimicrobial activity beyond its global regulatory role. These findings establish *Pseudomonas* sp. MUP55 as a taxonomically novel, mechanistically characterized biocontrol agent with potential for sustainable agriculture.

## 1. Introduction

Plant pathogens pose a significant threat to global food security, necessitating the development of sustainable control strategies. Among potential biocontrol agents, the genus *Pseudomonas*, first proposed by Migula in 1894 [1], has emerged as particularly promising due to its diverse antimicrobial capabilities and widespread distribution in agricultural ecosystems [2,3,4,5,6]. Members of this genus exhibit remarkable metabolic and genetic heterogeneity, with DNA G + C contents ranging from 58 to 69 mol% [3,7], and produce various bioactive compounds, including virulence factors, pigments, and motility-enhancing agents [8,9,10].

Within *Pseudomonas*, the *P. fluorescens* species group has garnered particular attention for its plant-beneficial properties [11,12]. This group comprises numerous strains that demonstrate robust biocontrol activities through the production of secondary metabolites, including antimicrobial compounds, siderophores, and various enzymes. Notable examples include *P. simiae* WCS417, which effectively suppresses soil-borne pathogens like *Gaeumannomyces graminis* var. *tritici* and *Fusarium oxysporum* [13,14,15,16], and *Pseudomonas lactis* SS101, which produces cyclic lipopeptide surfactants effective against various plant pathogens [13,14,15,16].

Recent advances in genomic technologies have revealed considerable complexity within the *P. fluorescens* species group taxonomy [11]. Current classification recognizes distinct subgroups based on multilocus phylogenetic analyses, yet rapid genome sequencing efforts continue to uncover novel diversity [11]. This expanding genomic landscape, coupled with frequent lack of species assignment and potential misclassification of isolates, has highlighted the need for comprehensive characterization of newly isolated strains, particularly those exhibiting promising biocontrol properties.

Cyclic lipopeptides (CLPs) are among the most important bioactive compounds in *Pseudomonas*-mediated biocontrol. Massetolide A, produced by *P. lactis* SS101, stands out as a particularly important CLP. It exhibits potent antimicrobial activity against various phytopathogens, promotes induced systemic resistance in plants, and contributes to bacterial motility and biofilm formation [17,18]. The gene cluster responsible for Massetolide biosynthesis was first identified in *P. lactis SS101* [19] and has subsequently been documented in numerous other beneficial *Pseudomonas strains* [18,20], highlighting its evolutionary conservation and ecological significance. Production of Massetolide A is primarily regulated through the Gac/Rsm signal transduction pathway, whereby GacS phosphorylates GacA, which then induces expression of small RNA genes (rsmY and rsmZ), sequestering translational repressors of key biosynthetic regulators [18,20].

Beyond the Gac/Rsm pathway, recent investigations have identified alternative quorum-sensing-like systems in various *Pseudomonas* strains. The *pvf* gene cluster, comprising a non-ribosomal peptide synthetase (NRPS, *pvfC*), a diiron N-oxygenase (*pvfB*), and two genes of unknown function (*pvfA* and *pvfD*), has been implicated in virulence and secondary metabolite production in *Pseudomonas entomophila* [21,22]. Homologous clusters in *Burkholderia cenocepacia* H111 (the *ham* cluster) produce valdiazen, a valinol-containing diazeniumdiolate that signals the expression of over 100 genes [23]. In *Pseudomonas syringae* pv. *syringae*, a homologous cluster produces leudiazen, which regulates mangotoxin production [24]. These studies have focused primarily on regulatory roles, while direct antimicrobial functions have remained largely unexplored, particularly in beneficial biocontrol strains.

In this study, we report the first comprehensive characterization of *Pseudomonas* sp. MUP55, and our data support the classification of *Pseudomonas* sp. MUP55 as a distinct species within the *P. fluorescens* group. Through a combination of genomic, metabolomic, phenotypic, and transcriptomic approaches, we identify Massetolide A as the primary bioactive compound and characterize its biosynthetic gene cluster; show the critical role of the GacA protein in Massetolide production through mutant analysis; demonstrate direct pathogen suppression; and uncover the dual role for the *pvf* cluster in the global regulation of specialized metabolism and in influencing secreted growth-inhibitory activity. The integrated dataset provides a comprehensive analysis of a beneficial *Pseudomonas* species.

## 2. Results

### 2.1. Genomic Characterization of Pseudomonas sp. MUP55

**Figure 1:**
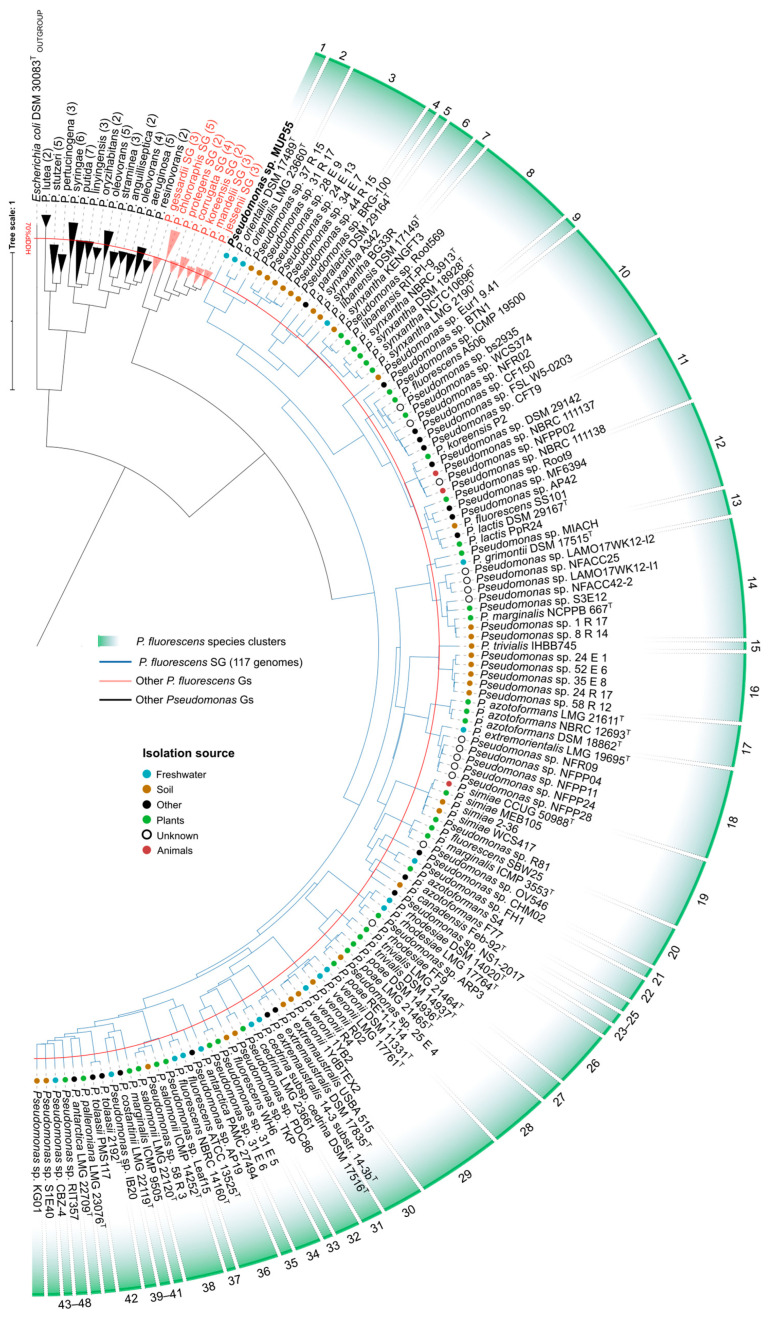
Phylogenetic tree of *Pseudomonas fluorescens* species clusters and related *Pseudomonas* groups. The circular tree represents 117 P. *fluorescens SG* (species group) genomes, along with other *P. fluorescens* and *Pseudomonas* groups. Branch colors indicate species classification: *P. fluorescens* SG (dark blue), other *P. fluorescens* groups (light blue), and other *Pseudomonas* groups (black). Colored dots at branch tips denote isolation sources: fresh water (blue), soil (brown), plants (green), animals (red), other (black), and unknown (white). The superscript T indicates type strains. The tree demonstrates the genetic diversity within *P. fluorescens* and its adaptation to various environmental niches. The scale bar represents genetic distance.

Our phylogenomic analysis establishes *Pseudomonas* sp. MUP55 as a species within the *P. fluorescens* group, with dDDH values significantly below the 70% threshold when compared to all 117 analyzed genomes. The online tool AutoMLST [25] was used to identify the closest species to *Pseudomonas* sp. MUP55. Among the top 997 genomes, we calculated intergenomic distances using the GBDP [26] algorithm, with the genome of *Pseudomonas* sp. MUP55 as a query, to select closely related genomes. Our findings indicate that a distance threshold of 0.138 unambiguously separates genomes, including *Pseudomonas* sp. MUP55, assigned to the *P. fluorescens* species group (SG) (Supplementary Table S1), resulting in 117 genomes putatively belonging to the *P. fluorescens* SG. These genomes were further analyzed to calculate pairwise GBDP intergenomic distances, corroborating their phylogenomic subgroup classification. The resulting phylogenomic tree (Figure 1; Supplementary Table S2) shows that the *P. fluorescens* SG forms a monophyletic group within the *P. fluorescens* species complex, with its closest neighboring subgroup being the *P. gessardii* SG. This phylogeny is consistent with previous analyses of the *P. fluorescens* species complex [27], but substantially expands the number of genomes belonging to the *P. fluorescens* SG.

The clustering analysis of intergenomic distances (Figure 1; Supplementary Table S2) reveals that at the species level (dDDH ≥ 70%, corresponding to a distance threshold of T = 0.036), the *P. fluorescens* SG comprises 48 distinct species clusters. Notably, 22 of these clusters are represented by only a single genome, including *Pseudomonas* sp. MUP55, indicating a potential for uncovering further diversity within this group. Additionally, the phylogenetic analysis of the *P. fluorescens* SG identified instances where genomes have been incorrectly assigned to SG clusters or remain unclassified. These genomes require reclassification or formal naming, especially for novel species. The strain *Pseudomonas* sp. MUP55 exhibited low relatedness with all other genomes in the *P. fluorescens* SG, with dDDH values significantly below the 70% species delineation threshold (Figure 1; Supplementary Table S2).

To further support this classification, we calculated ANI and dDDH values, together with genome size and GC content, for *Pseudomonas* sp. MUP55 against its 17 closest type strains identified via TYGS (Supplementary Table S3). The closest relative, by both metrics, was *Pseudomonas orientalis* DSM 17489 (dDDH formula d4 = 41.3%, ANI = 91.9%), both well below the recognized species thresholds (dDDH ≥ 70%; ANI ≥ 95–96%), corroborating that *Pseudomonas* sp. MUP55 represents a distinct species.

### 2.2. LC-MS and Molecular Networking Identify Massetolide A/D as the Leading Candidate Bioactive Compound

**Figure 2:**
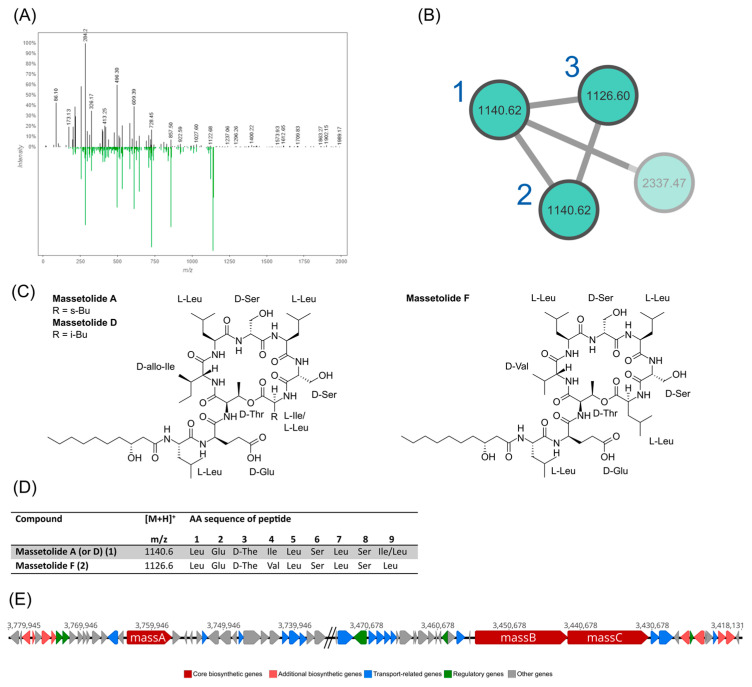
Massetolide production in *Pseudomonas* sp. MUP55. (**A**) LC-MS results from the supernatant compared to a Massetolide A reference, using GNPS. Mirror plot comparing the experimental spectrum (top) with the matched reference library spectrum (bottom, inverted); shared fragment ion *m*/*z* peaks indicate the spectral similarity used to assign compound identity. (**B**) The molecular network of identified Massetolides; nodes 1 and 2 both match library spectra for Massetolide A/D (isomers not distinguished by available MS/MS data), and node 3 represents Massetolide F. The blurred node is for an unidentified compound. (**C**) Chemical structures of Massetolide A and D, illustrating the Ile/Leu substitution that distinguishes the two isomers (not resolved in this analysis). (**D**) Table showing the mass-to-charge ratio ([M + H]^+^) and amino acid sequence of Massetolide A (or D) and Massetolide F. (**E**) The biosynthetic gene cluster and structural analysis of Massetolides in *Pseudomonas* sp. MUP55. Genomic organization of the Massetolide biosynthetic gene cluster in *Pseudomonas* sp. MUP55. The cluster includes core biosynthetic genes (massA, massB, and massC), additional biosynthetic genes, transport-related genes, regulatory genes, and other genes.

To identify the metabolite responsible for *Pseudomonas* sp. MUP55’s antimicrobial activity, the culture supernatant was characterized by mass spectrometry and molecular networking. LC-MS and GNPS molecular networking identified two related nodes matching library reference spectra for Massetolide A/D (*m*/*z* = 1140.6 [M + H]^+^; the Ile/Leu isomers could not be distinguished by the available MS/MS fragmentation data), together with a minor feature matching Massetolide F (*m*/*z* = 1126.6) (Figure 2A,B), present in the supernatant of *Pseudomonas* sp. MUP55 (Figure 2C,D). Massetolide A is known for its biocontrol properties, particularly against phytopathogens [19,28]; given the mass and fragmentation similarity between Massetolide A and D, we cannot rule out that both nodes represent the same isomer or a mixture of the two.

The biosynthesis of Massetolide A involves non-ribosomal peptide synthetases (NRPSs) encoded by a specific gene cluster in *Pseudomonas* strains [19,28]. Genomic analysis of *Pseudomonas* sp. MUP55 using antiSMASH [29,30] identified a biosynthetic gene cluster (BGC) containing the core biosynthetic genes massA, massB, and massC, along with transport-related and regulatory genes (Figure 2E). This BGC matches previously reported Massetolide A BGCs from *P. lactis* SS101 and related strains. The detection of Massetolide F alongside the ambiguous Massetolide A/D signal is consistent with some flexibility in the NRPS machinery, though we cannot confirm from the current data whether distinct A and D isomers are genuinely co-produced.

### 2.3. GacA Regulates Massetolide Production and Biocontrol Efficacy

**Figure 3:**
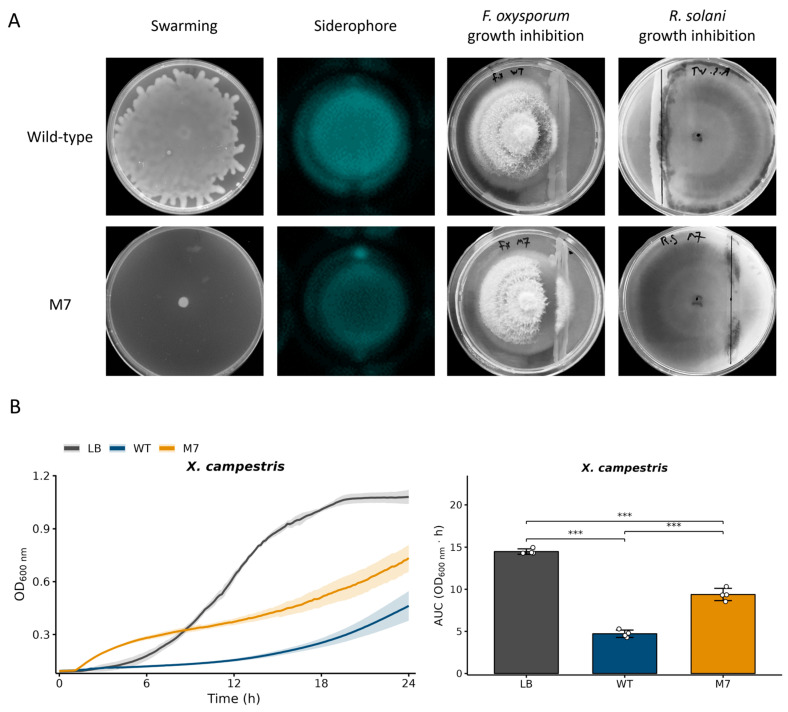
Comparative analysis of wild-type *Pseudomonas* sp. MUP55 and the mutant M7 strain. (**A**) Phenotypic characterization of wild-type (WT) and M7 mutant strains. Swarming motility assay on 0.5% LB agar plates after 24 h. Siderophore production visualized under UV light. Inhibition of *F. oxysporum* growth after 6 days. Inhibition of *R. solani* growth after 4 days. The M7 mutant shows reduced swarming motility, decreased siderophore production, and diminished antifungal activity compared to the WT strain. The differences between WT and M7 were significant for both pathogens (Welch’s *t*-test, *p* < 0.0001. (**B**) Growth of *X. campestris* in the presence of cell-free supernatants from the WT strain, the M7 mutant, or LB-only control (*n* = 4 biological replicates per condition); OD_600_ nm was monitored over 24 h. Lines show the mean and shaded ribbons the SD. Total growth per replicate was summarized as the area under the curve (AUC) and compared by Welch’s *t*-test with Holm correction for the three pairwise comparisons within each pathogen (*** *p* < 0.001). Per-panel significance annotations show WT vs. control, M7 vs. control, and WT vs. M7.

To identify regulatory determinants of biocontrol activity, swarming-deficient mutants were generated by successive subculturing on swarming plates over five cycles [31]. After five cycles, cells were spread on 1.5% LB agar, and colonies were screened for loss of swarming. Successive subculturing yielded ten swarming-attenuated mutants (M1–M10) exhibiting a range of swarming phenotypes, from partial reduction to complete loss. Mutant M7 was selected for in-depth characterization based on the complete absence of swarming motility (0% of WT diameter (Figure 3A)). Siderophore production in M7, quantified by fluorescence intensity at 405 nm, was 63% of the WT intensity (Figure 3A).

Antifungal activity revealed the most striking differences. Based on three biological replicates (SD = 0.5% for all conditions), the WT achieved 42% inhibition of radial growth of *F. oxysporum* and 50% inhibition of *Rhizoctonia solani*. In contrast, the M7 mutant showed only 12.5% inhibition against *F. oxysporum* and complete loss of activity against *R. solani* (0%; Figure 3A). Welch’s two-tailed *t*-tests (df = 4) confirmed both differences were highly significant (*F. oxysporum*: t = 72.26, *p* = 2.2 × 10^−7^; *R. solani*: t = 122.47, *p* = 2.7 × 10^−8^). Antibacterial activity was evaluated using cell-free supernatants of the WT and M7 strains against *Xanthomonas campestris*, with LB-only as the non-inhibitory control (Figure 3B). Against *X. campestris,* the supernatants were clearly separable. WT supernatant reduced AUC by 67%, while M7 supernatant produced only a 35% reduction, with the WT–M7 difference itself highly significant (Figure 3B). M7 therefore exhibits a substantial but partial loss of inhibitory activity against *X. campestris*.

LC-MS analysis confirmed complete absence of Massetolide A and related variants in M7 supernatants. Whole-genome sequencing of M7 identified a P58L point mutation in GacA, the response regulator of the GacS/GacA two-component system and the only variant detected relative to the wild-type genome [31]. This single amino acid change in the signal receiver domain of GacA is sufficient to abolish Massetolide biosynthesis, establishing GacA as an essential positive regulator of Massetolide production in *Pseudomonas* sp. MUP55.

### 2.4. Pseudomonas sp. MUP55 Supernatant Exhibits Bacteriostatic Activity Against E. coli

**Figure 4:**
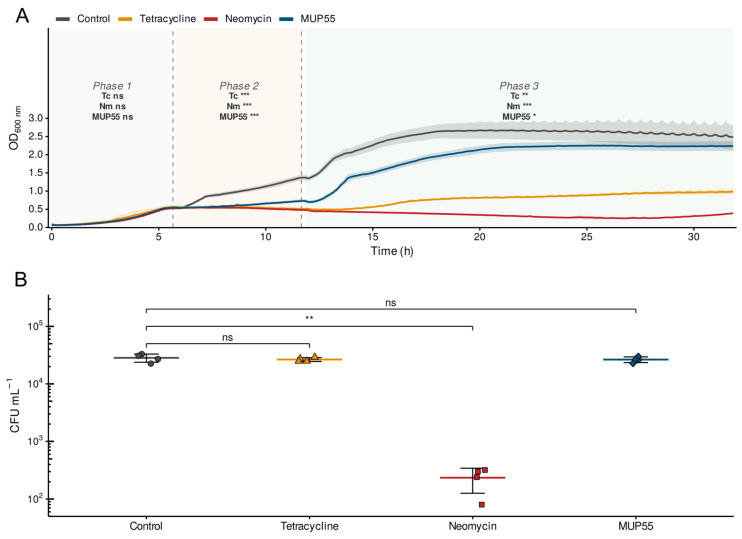
*Pseudomonas* sp. MUP55 supernatant is bacteriostatic against *E. coli*. (**A**) Continuous OD_600_ nm trajectory across initial growth (Phase 1), treatment exposure (Phase 2), and post-wash recovery (Phase 3) for *E. coli* in LB only (control), tetracycline (10 µg/mL, bacteriostatic reference), neomycin (50 µg/mL, bactericidal reference), or sterile *Pseudomonas* sp. MUP55 supernatant. Curves show mean ± SD (*n* = 4 replicates); traces were normalized across phases for the dilution and wash steps to produce a continuous trajectory. Dashed vertical lines mark phase transitions; per-phase comparisons (Tc, Nm, and MUP55 vs. control) by Welch’s *t*-test on AUC with Holm correction are shown above each phase (*** *p* < 0.001, ** *p* < 0.01, * *p* < 0.05, and ns = not significant). (**B**) CFU/mL recovered on LB agar after the wash step (*n* = 4); mean is shown as the horizontal crossbar with SD whiskers, with individual replicates overlaid. Welch’s *t*-test vs. control with Holm correction: ** *p* < 0.01, and ns = not significant. Tetracycline and MUP55 supernatant produce CFU count indistinguishable from control, while neomycin reduces viability by ~3 log10, distinguishing the bacteriostatic mode of action of *Pseudomonas* sp. MUP55 supernatant from the bactericidal control.

Having identified Massetolide A/D as the leading candidate antimicrobial determinant of *Pseudomonas* sp. MUP55, we next characterized the mode of this inhibition. To determine whether the inhibitory activity of *Pseudomonas* sp. MUP55 supernatant is bactericidal or bacteriostatic, a distinction rarely investigated in biocontrol studies, a three-phase optical density assay was combined with a post-treatment colony-forming unit (CFU) recovery readout, with tetracycline (bacteriostatic [32]) and neomycin (bactericidal [33]) included as reference antibiotics [34]. *Escherichia coli* was first grown in LB to mid-exponential phase (Phase 1); diluted into fresh medium containing either LB only (control), tetracycline, neomycin, or sterile *Pseudomonas* sp. MUP55 supernatant; and incubated for a further 6 h (Phase 2, treatment exposure). Cells were then pelleted, washed in fresh LB to remove residual treatment, standardized to OD_600_ nm = 0.2, and (i) returned to fresh LB to monitor regrowth (Phase 3, post-wash recovery) and (ii) plated on LB agar for CFU enumeration (Figure 4A).

During Phase 1, all four arms grew identically, confirming a uniform starting culture. During Phase 2, growth was significantly suppressed in all three treatments relative to the control, establishing that *Pseudomonas* sp. MUP55 supernatant inhibited active growth to an extent comparable to both reference antibiotics. The mean Phase 2 AUC for MUP55-treated cultures was 3.59 ± 0.21 versus 5.72 ± 0.34 for control, corresponding to a 37% reduction in total growth.

The post-wash readouts then resolved the static-versus-cidal question. Following the wash, neomycin-treated cultures yielded 235 CFU/mL, a 3-log reduction relative to control (2.83 × 10^5^ CFU/mL), confirming bactericidal activity. In contrast, both tetracycline-treated (2.65 × 10^5^ CFU/mL) and MUP55-treated (2.65 × 10^5^ CFU/mL) cultures recovered CFU counts statistically indistinguishable from the control (Figure 4B), indicating that cells exposed to *Pseudomonas* sp. MUP55 supernatant remained viable. Consistent with this, in Phase 3, the MUP55-exposed cells resumed growth and reached a final OD_600_ nm of 2.25, approaching the control endpoint of 2.65, whereas neomycin-treated cells failed to recover and tetracycline-treated cells exhibited only partial recovery within the observation window. Together, the equivalence in CFU recovery and the resumption of exponential growth after washout demonstrate that *Pseudomonas* sp. MUP55 supernatant exerts a bacteriostatic rather than bactericidal effect on *E. coli*.

### 2.5. pvfC Contributes to Secreted Growth-Inhibitory Activity

**Figure 5:**
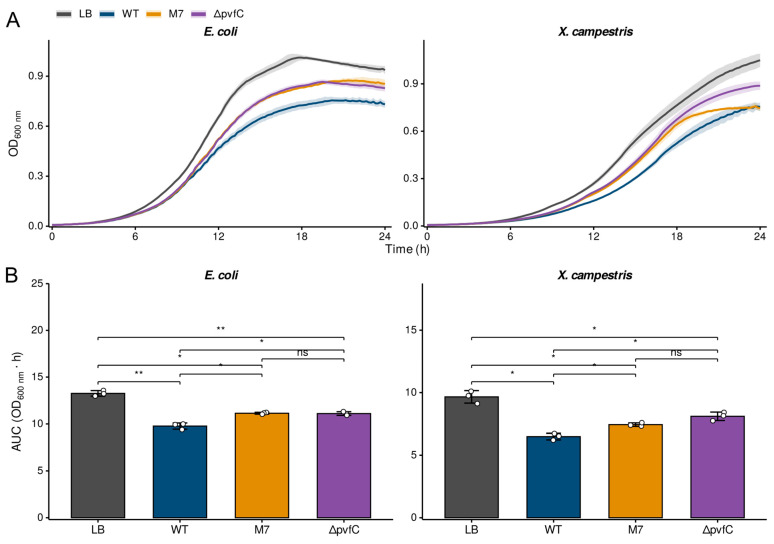
Cell-free supernatants of WT, M7, and Δ*pvfC* differentially inhibit the growth of *E. coli* and *X. campestris*. Cell-free supernatants of WT MUP55, the gacA-P58L mutant M7, and WT ΔpvfC were diluted 1:1 *v*/*v* into fresh LB, inoculated with *E. coli* or *X. campestris*, and growth was monitored at OD_600_ nm every 10 min over 24 h in a plate reader at 28 °C with continuous shaking. (**A**) Mean OD_600_ nm trajectories; shaded ribbons show ±SD. (**B**) Total growth per replicate, summarized as the trapezoidal AUC of the OD_600_ nm curve over 0–24 h; bars show mean ± SD with individual replicate values overlaid. The six pairwise supernatant comparisons within each target were tested by Welch’s *t*-test with Holm correction for multiple comparisons (** *p*_adj < 0.01, * *p*_adj < 0.05, and ns = not significant).

The incomplete loss of antibacterial activity in M7, despite the complete absence of Massetolide, indicated that additional secreted compounds contribute to inhibition. To dissect the contribution of *pvfC*-cluster products to the inhibitory secretome of *Pseudomonas* sp. MUP55, growth of *E. coli* and *X. campestris* was monitored over 24 h in 1:1 *v*/*v* dilutions of cell-free supernatants of WT, M7, and *ΔpvfC* into LB, alongside an LB-only control (Figure 5). Total growth per replicate was summarized as the trapezoidal area under the OD_600_ nm curve (AUC, 0–24 h). All three supernatants significantly inhibited *E. coli* growth relative to the LB control (Figure 5A,B), consistent with the bacteriostatic effect characterized in the preceding section. WT supernatant was the most potent, while M7 supernatant and Δ*pvfC* supernatant each produced significantly less inhibition than WT (WT vs. M7 *p*_adj = 0.026; WT vs. ΔpvfC *p*_adj = 0.022). M7 and Δ*pvfC* supernatants were statistically indistinguishable from each other (*p*_adj = 0.80). The combined effect, that loss of Massetolide (in M7) or loss of *pvfC* (in Δ*pvfC*) reduces inhibitory activity to a similar partial extent, while WT remains substantially more potent than either single mutant, is consistent with Massetolide and pvfC-cluster products contributing overlapping but distinct antimicrobial activities against *E. coli. X. campestris* showed the same qualitative pattern (Figure 5A,B). All three supernatants inhibited *X. campestris* growth relative to LB (WT 32.8%, M7 23.0%, and *ΔpvfC* 16.1% reduction in AUC; all *p*_adj < 0.05). WT supernatant was significantly more inhibitory than M7 (*p*_adj = 0.045) and Δ*pvfC* (*p*_adj = 0.017); the M7-versus-Δ*pvfC* comparison did not reach significance after correction (*p*_adj = 0.060). As for *E. coli*, the WT supernatant exceeded both single-mutant supernatants in inhibitory activity, and neither single mutation abolished inhibitory activity entirely.

### 2.6. The pvf Signaling Pathway Is Associated with Broad Transcriptional Changes

**Figure 6:**
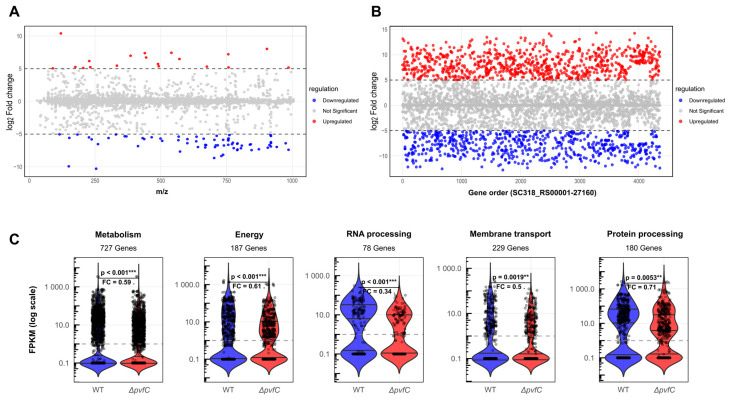
Metabolomic and transcriptomic analysis comparing WT and *ΔpvfC* strains. (**A**) Comparative cell-free metabolites WT and *ΔpvfC* strains. Scatter plot of the log_2_ fold change (*ΔpvfC*/WT) (y-axis) between the mean intensity of the metabolites and the *m*/*z* (x-axis). Each feature is defined as an ion with a unique *m*/*z* and retention time; often, a compound will be represented by multiple features. (**B**) Differential gene expression analysis comparing WT and *ΔpvfC* strains. The scatter plot displays log_2_ fold change (*ΔpvfC*/WT) (y-axis) for each gene (x-axis, ordered by gene ID from SC318_RS00001 to SC318_RS27160). Each point represents an individual gene, with positive values indicating higher expression in the *ΔpvfC* mutant relative to WT strain, and negative values indicating lower expression. The dashed horizontal line at y = 0 represents no change in expression between conditions. (**C**) Different expression between WT and *ΔpvfC* strains across five functional categories. Metabolism, energy, RNA processing, membrane transport, and protein processing, *** *p* < 0.001, ** *p* < 0.01.

Comparative metabolomics (XCMS) and transcriptomics comparing WT and Δ*pvfC* strains revealed extensive transcriptional changes associated with loss of the *pvf* signal (Figure 6). PvfC itself is an enzyme required for biosynthesis of the pvf signaling molecule; the broad transcriptional impact described below reflects the downstream consequences of losing this signal, which may include loss of the signal itself, secondary physiological effects, altered growth state, and indirect regulatory cascades, rather than direct transcriptional regulation by PvfC. Of 1413 metabolite features detected, 70 were downregulated and 19 upregulated in Δ*pvfC* (Figure 6A); these features were flagged using an uncorrected, exploratory significance threshold and represent candidates for follow-up rather than statistically confirmed differentially abundant metabolites. Of 4359 genes with measurable expression, 848 showed upregulation and 647 showed downregulation in Δ*pvfC* compared to WT, with log_2_ fold changes ranging from −12.80 to +14.33 (Figure 6B).

Functional categorization across five major cellular function categories revealed statistically significant expression differences (Figure 6C). The most dramatic changes were observed in RNA processing genes (78 genes), where expression in the Δ*pvfC* strain was reduced to approximately one-third of WT levels. Significant downregulation was also observed in metabolism (727 genes), energy metabolism (187 genes), membrane transport (229 genes), and protein processing (180 genes) categories, indicating a broad regulatory impact of *pvfC* on essential cellular functions.

### 2.7. PvfC Differentially Regulates Dual Siderophore Systems

**Figure 7:**
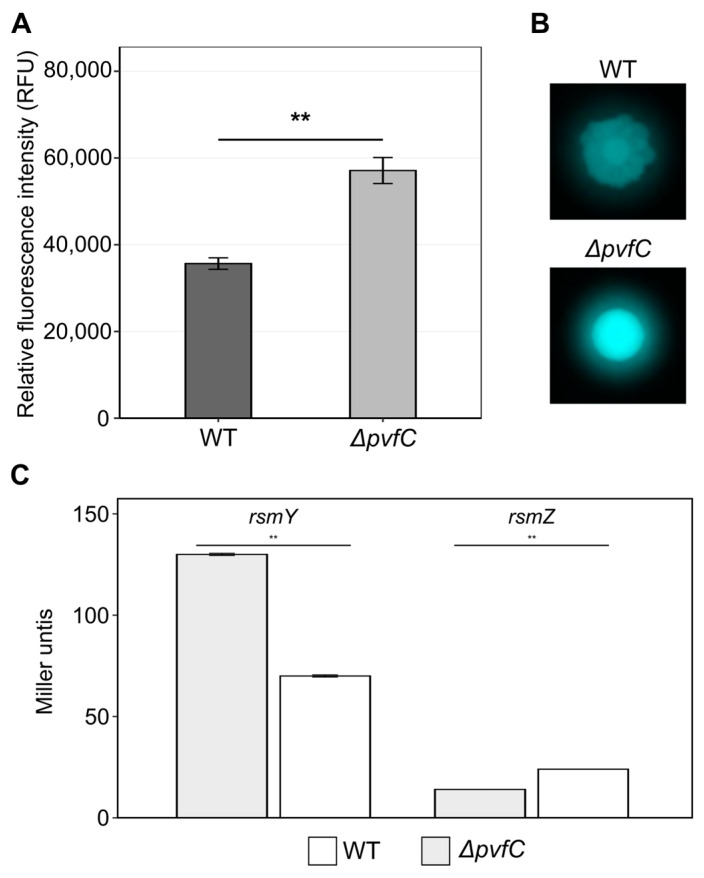
Relative fluorescence intensity (RFU) comparison between the WT and Δ*pvfC* strains, and the expression of *rsmY* and *rsmZ* promoters in a β-galactosidase assay. (**A**) The RFU between the WT and Δ*pvfC* strains. (**B**) Fluorescence intensity comparison between the WT and Δ*pvfC* colonies after 48 h of growth in LB agar plate. (**C**) The expression of *rsmY* and *rsmZ* promoters in a β-galactosidase assay. The white and the gray bars represent the WT and Δ*pvfC*, respectively. ** *p* < 0.05 by ANOVA.

A striking example of *pvfC*’s regulatory role was observed in siderophore production. The Δ*pvfC* mutant exhibited significantly increased fluorescence compared to WT (Figure 7A,B), indicating altered siderophore production. Genomic analysis confirmed that *Pseudomonas* sp. MUP55 possesses two distinct siderophore biosynthetic pathways: NRPS-independent (producing a triabactin-like compound [35]), and the NRPS-dependent pyoverdine system (Table 1).

Transcriptomic profiling showed that PvfC reciprocally regulates the two siderophore systems. The triabactin-like gene *trbA* was strongly and significantly induced in Δ*pvfC* (log_2_FC +11.0, *p*adj = 1.3 × 10^−11^); *trbC* showed a similarly large nominal increase (log_2_FC +11.2) that narrowly missed conventional significance after correction (*p*adj = 0.053), whereas the core pyoverdine biosynthesis and uptake genes were repressed in Δ*pvfC* (pyoverdine NRPS −8.1, fpvA −6.6, PvdM −5.2 log_2_FC; only minor accessory genes such as the PvdJ/PvdD/PvdP-like protein were elevated, +1.7). Because pyoverdine fluorescence reports the iron-free (apo) form and is quenched upon Fe^3+^ binding, the increased fluorescence of the mutant most likely reflects an enlarged apo-pyoverdine pool rather than greater pyoverdine synthesis: reduced expression of the ferripyoverdine receptor fpvA would limit reuptake of iron-loaded pyoverdine and favor extracellular accumulation of the fluorescent apo form, while the post-transcriptional action of the pvf/Gac–Rsm system means pyoverdine output need not track transcript abundance. PvfC therefore appears to reshape the iron-acquisition strategy in *Pseudomonas* sp. MUP55, derepressing the triabactin-like system while repressing pyoverdine biosynthesis and uptake, rather than uniformly increasing siderophore production (Table 1).

### 2.8. PvfC Modulates the Massetolide Regulatory Network and Uncouples rsmY and rsmZ

Transcriptomic analysis revealed nominal expression changes in several genes associated with the Massetolide biosynthetic regulatory network (Table 2), although most did not reach statistical significance after correction for multiple testing (*p*adj < 0.05). The transcriptional regulators *luxR1* (log_2_FC = 1.68; *p*adj = 0.61) and *luxR2* (log_2_FC = 8.97; *p*adj = 0.12) trended toward upregulation in the Δ*pvfC* strain, and the anti-sigma factor gene *prtR* trended toward downregulation (log_2_FC = −10.69; *p*adj = 0.064), approaching but not reaching significance. If genuine, these trends would be consistent with a model in which reduced *prtR* expression contributes to *luxR2* upregulation, given the previously reported regulatory relationship between *prtR* and *luxR2* [18]; however, this should be treated as a hypothesis for future validation rather than an established regulatory link.

To further investigate effects on the Gac/Rsm regulatory pathway, expression of rsmY and rsmZ was measured using β-galactosidase reporter assays (promoter::lacZ fusions). Remarkably, rsmY expression increased significantly in the Δ*pvfC* mutant compared to WT, while rsmZ expression decreased significantly (Figure 7C). This uncoupling of two typically co-regulated small RNAs represents a novel regulatory mechanism by which *pvfC* influences the Gac/Rsm pathway, suggesting that PvfC interacts with specific regulatory components that differentially target rsmY and rsmZ. Of the Massetolide NRPS structural genes, *massB* was significantly downregulated in Δ*pvfC* (log_2_FC = −2.38, *p*adj = 0.042), while *massA* showed a large nominal fold-change (log_2_FC = 6.06) that could not be reliably assessed due to exclusion by independent filtering, and *massC* showed no notable change. The significant change in *massB* indicates that PvfC’s influence on the Massetolide biosynthetic gene cluster may not be limited to upstream signaling components as previously proposed, and that a direct or indirect effect on at least one structural gene cannot be excluded (Table 2).

## 3. Discussion

The isolation of *Pseudomonas* sp. MUP55 from rainwater in Western Australia and its classification as a distinct species within the *P. fluorescens* SG highlights the untapped microbial diversity present in non-soil environmental sources. The singleton status of *Pseudomonas* sp. MUP55 at the dDDH ≥ 70% threshold, alongside 21 other singleton clusters among the 48 species clusters identified, highlights how substantially the true diversity of this group remains uncharted.

The identification of Massetolide A/D as the leading candidate bioactive compound(s), supported by LC-MS, GNPS molecular networking, and antiSMASH BGC annotation, is consistent with the established role of this cyclic lipopeptide class as a key antimicrobial in plant-beneficial *Pseudomonas strains* [17,18]. The two isomers could not be distinguished by the available MS/MS fragmentation data; this, together with the detection of a minor Massetolide F feature, is consistent with some flexibility in the NRPS machinery, though we cannot confirm from the current data whether both isomers are genuinely co-produced or represent a single compound matched ambiguously to both library entries. The bacteriostatic, rather than bactericidal, mode of action demonstrated against *E. coli* is a distinction rarely investigated in biocontrol studies; whether this holds for other pathogens remains an important open question.

The GacA P58L mutation, sufficient to abolish Massetolide biosynthesis entirely and substantially reduce biocontrol efficacy, confirms GacS/GacA as an essential regulatory axis for CLP production in *Pseudomonas* sp. MUP55. The proline substitution at position 58 of the receiver domain likely disrupts the conformational change required for downstream signal transduction, consistent with spontaneous loss-of-function *gac* mutations documented in *P. protegens* under swarming selection [30]. The partial retention of antibacterial activity against *X. campestris* in M7 despite complete Massetolide loss indicates that additional GacA-independent antimicrobial compounds are produced by *Pseudomonas* sp. MUP55, warranting further chemical characterization. As M7 arose by spontaneous mutation and was not genetically complemented, attribution of these phenotypes to the P58L substitution, although it was the only genomic change detected relative to the wild type, would be strengthened in future work by complementation or by a defined Massetolide-biosynthesis mutant. A defined biosynthesis mutant would also separate the lipopeptide’s direct contribution from the broader GacA regulon, since the antifungal activity was scored in a live dual-culture format that additionally reflects siderophore, volatile, and competitive effects, whereas the antibacterial activity was assessed with cell-free supernatant. The Δ*pvfC* mutant used throughout this study was similarly not genetically complemented; as with M7, causal attribution of *pvfC*-dependent phenotypes should be treated as correlative pending complementation or equivalent confirmatory evidence.

A significant contribution of this study is evidence that the *pvf* cluster of *Pseudomonas* sp. MUP55 contributes both to the regulation of specialized metabolism and to secreted growth-inhibitory activity. While prior work on *pvf* homologues in *P. entomophila* [21,22], *B. cenocepacia* [23], and *P. syringae* [24] has focused *pr*imarily on regulatory signaling roles, the partial but reproducible reduction in inhibitory potency of Δ*pvfC* supernatants against both *E. coli* and *X. campestris* indicates that *pvfC* contributes to the secreted antimicrobial phenotype of *Pseudomonas* sp. MUP55, in addition to its broader regulatory functions; we have not isolated or directly tested a pvf-derived metabolite for antimicrobial activity, and a more parsimonious interpretation, that *pvfC* deletion alters production of other secreted inhibitory metabolites such as Massetolide A/D, cannot be excluded. Disentangling the direct biosynthetic contribution from indirect effects mediated by *pvfC*’s global regulatory role will require dedicated biochemical characterization of pvf-dependent metabolites in future work.

The global transcriptomic impact of *pvfC* deletion, affecting over a third of expressed genes, places the *pvf* cluster in a regulatory tier above most characterized secondary metabolite gene clusters, although we note that most of the individual regulatory genes examined in this network did not themselves reach statistical significance after correction for multiple testing (Table 2), and the effect of *pvfC* deletion on this specific regulatory cascade should be interpreted as a trend rather than an established result. Most striking is the uncoupling of rsmY and rsmZ, two small RNAs generally treated as functionally redundant outputs of the Gac/Rsm pathway [18,20]. The opposing direction of their expression changes in Δ*pvfC*, alongside a nominal, though not statistically significant, downregulation of the anti-sigma factor *prtR* (log_2_FC = −10.69, *p*adj = 0.064) and a corresponding nominal upregulation of *luxR2* (log_2_FC = 8.97, *p*adj = 0.12), is consistent with a model in which the *pvf* cluster interfaces with the Gac/Rsm cascade through a component that selectively influences *rsmZ* but not *rsmY* transcription; however, this specific link should be treated as a hypothesis for future validation rather than a demonstrated mechanism. Because the rsmY/rsmZ uncoupling itself rests on promoter–reporter fusions, direct quantification of the two small RNAs would be valuable to confirm it, alongside validation of the *prtR*/*luxR1*/*luxR2* trend. Notably, of the Massetolide biosynthetic genes, *massB* was significantly downregulated in Δ*pvfC* (log_2_FC = −2.38, *p*adj = 0.042), indicating that *pvfC*’s influence on the Massetolide gene cluster may not be confined to upstream signaling components as initially proposed, and that a direct or indirect effect on at least one structural gene cannot be excluded. The bidirectional control of dual siderophore systems, simultaneously suppressing the NRPS-independent pathway while sustaining the NRPS-dependent pathway, further shows how the *pvf* cluster coordinates competing iron acquisition strategies, likely optimizing competitive fitness under the iron-limiting conditions of the rhizosphere.

Several limitations should be considered when interpreting these findings. Neither the GacA P58L mutant (M7) nor the Δ*pvfC* mutant was genetically complemented, so phenotypic attributions for both remain correlative rather than causal. Evidence for the antimicrobial contribution of the *pvf* cluster and of Massetolide A/D is indirect, relying on loss-of-function phenotypes rather than direct testing of purified compounds. The rsmY/rsmZ uncoupling and the prtR/luxR1/luxR2 regulatory trend rest on promoter–reporter and transcriptomic data, respectively, that would benefit from direct transcript-level validation. Finally, taxonomic characterization of *Pseudomonas* sp. MUP55, while supported by dDDH, ANI, and phenotypic/chemotaxonomic data, has not yet been extended to a full comparative phenotypic panel beyond antimicrobial traits.

## 4. Materials and Methods

### 4.1. Culture Conditions

*Pseudomonas* sp. MUP55 and *X. campestris* pv. *campestris* (WAC14181) were routinely cultured in LB medium at 28 °C (200 rpm). *E. coli* DH5α (New England Biolabs, Notting Hill, VIC, Australia) was grown in LB medium at 37 °C (200 rpm) for routine culture; for the antimicrobial, bacteriostatic, and supernatant-inhibition assays, it was incubated at 28 °C to match the other test strains. *F. oxysporum* (WAC10262) and *R. solani* (WAC14439) were cultured on PDA (Becton Dickinson Difco, Macquarie Park, NSW, Australia) at 28 °C. *X. campestris*, *F. oxysporum*, and *R. solani* isolates were obtained from the Western Australian culture collection (WAC), Department of Primary Industries and Regional Development (DPIRD) Diagnostics and Laboratory Services, in accordance with Australian biosecurity restrictions on the importation of plant-pathogenic strains. All experiments were conducted in triplicate unless otherwise stated.

### 4.2. Genome Sequencing and Comparative Genomics

High-molecular-weight genomic DNA was extracted using the Qiagen MagAttract HMW DNA Kit (Qiagen, Clayton, VIC, Australia) from a logarithmic-phase culture, obtained by subculturing a saturated overnight culture and incubating for a further 6 h prior to extraction. ONT libraries were prepared according to the ONT 1D ligation library preparation protocol (SQK-LSK109) and sequenced with a FLO-MIN-106D flow cell (R9.4.1) on a MinION platform, yielding 337,900 reads (1.2 Gb total; mean read length ~3551 bp), corresponding to ~202× coverage of the 5,948,019 bp genome. Guppy v3.2.6 was used for base calling (read-pass-filter quality score cutoff = 7). Long reads were assembled using Flye v2.9 (10 iterations, default parameters), producing a single circular chromosome. BUSCO v5.3.2 assessed assembly completeness (99.9%). The whole-genome sequence was deposited in GenBank under accession CP138214, and raw reads under SRR26637682. ANIb was determined using FastANI [36], dDDH using GGDC formula 2 (https://ggdc.dsmz.de). Phylogenomic analyses used autoMLST [25] and GBDP [26] with *E. coli* DSM 30083^T^ as outgroup; trees were visualized in iTOL v6 [37].

### 4.3. Genome-Based Taxonomic Analysis

The autoMLST tool determined the 997 closest genomes to *Pseudomonas* sp. MUP55. Intergenomic distances were calculated using GBDP at http://ggdc.dsmz.de. Pairwise distances of the 117 putative *P. fluorescens* SG genomes, together with 81 genomes from outside the species group (198 in total), were transformed into a distance matrix and used to build a neighbor-joining phylogenomic tree using Shiny [38]. Additionally, Whole-genome-based species delimitation was additionally performed using the Type (Strain) Genome Server (TYGS; /tygs.dsmz.de) [39]. Closest type strain genomes were identified in two complementary ways: first, all genomes in the TYGS database were compared against the *Pseudomonas* sp. MUP55 genome via the MASH algorithm [40], and the ten type strains with the smallest MASH distances were selected; second, 16S rDNA gene sequences were extracted from the *Pseudomonas* sp. MUP55 genome, using RNAmmer v1.2 [41] and BLASTed v2.16.0 [42], against the 16S rDNA sequences of the 24,577 type strains in the TYGS database, from which the top 50 matches by bitscore were used to calculate precise intergenomic distances via the GBDP approach under the “coverage” algorithm and distance formula d5 [26], yielding a further ten closest type strains. Pairwise genome comparisons among the resulting set were conducted using GBDP under the “trimming” algorithm and distance formula d5 [26], with 100 distance replicates per comparison. Digital DDH values (formulae d0, d4, and d6) and confidence intervals were calculated using the recommended settings of the GGDC 4.0 [26]. ANI was calculated using FastANI [36] for the same comparator set. Species-level clustering used a 70% dDDH radius [26].

### 4.4. Phenotypic and Chemotaxonomic Characterization

Growth of *Pseudomonas* sp. MUP55was assessed in LB medium across a temperature range of 4–37 °C, at pH values from 5.0 to 8.0 in increments of 0.5, and at NaCl concentrations from 0 to 4% (*w*/*v*) in increments of 0.5%. Carbon source utilization and chemical sensitivity were assessed using Biolog GEN III MicroPlates (Biolog, Hayward, CA, USA), and assimilation and enzymatic activity were assessed using API 20NE strips (bioMérieux, Nürtingen, Germany). Cellular fatty acid, polar lipid, and respiratory quinone composition were determined by the Identification Service, Leibniz-Institut DSMZ (Braunschweig, Germany), using their standard chemotaxonomic identification protocols.

### 4.5. Bacteriostatic/Bactericidal Differentiation Assay

*E. coli* was grown in LB to mid-exponential phase (Phase 1, 6 h, 28 °C, 900 rpm) in a Varioskan LUX Multimode Microplate Reader (ThermoFisher, Scoresby, VIC, Australia) and then diluted into fresh medium containing one of four treatments: LB only (control), tetracycline (10 µg/mL, bacteriostatic reference), neomycin (50 µg/mL, bactericidal reference), or sterile *Pseudomonas* sp. MUP55 supernatant (1:1 *v*/*v* with LB; prepared by centrifugation at 10,000× *g* for 5 min followed by 0.22 µm filter sterilization). OD_600_ nm was recorded at 10 min intervals for a further 6 h (Phase 2, treatment exposure). Cells were then harvested by centrifugation (10,000× *g*, 2 min), washed twice with fresh LB to remove residual treatment, and the pellets resuspended and standardized to OD_600_ nm = 0.2. A 50 μL aliquot of each suspension was spread on LB agar and incubated for 18 h at 28 °C for CFU enumeration. The remaining suspensions were returned to the plate reader in fresh LB and monitored for a further 20 h (Phase 3, post-wash recovery). For visualization of continuous growth dynamics, OD_600_ nm traces were blank-subtracted, and per-replicate scaling factors were applied at each phase transition to compensate for the dilution and wash steps. The assay was performed in four biological replicates. Statistical comparisons were computed on the area under the raw, unscaled OD_600_ nm curve; the per-replicate scaling described above was applied for visualization only. Treatments were compared to control by Welch’s *t*-test with Holm correction for multiple comparisons, and CFU/mL counts were compared to control by the same procedure.

### 4.6. Successive Swarming and Mutant Generation

Successive swarming was performed as previously described [31]. Briefly, *Pseudomonas* sp. MUP55 was inoculated at the center of 0.5% LB agar plates. After 24 h, cells from the colony edge were transferred successively to new swarm plates for 5 cycles. After 5 cycles, cells were washed and spread on 1.5% LB agar. Colonies were screened for loss of swarming. For swarming motility assays, cultures grown for 18 h were spotted on 0.5% LB plates and incubated at 28 °C for 24 h. Siderophore production was assessed in 96-well plate format (200 µL culture per well), with relative fluorescence intensity measured at 405 nm using a Varioskan LUX plate reader; uninoculated medium served as a blank control for background fluorescence. The Δ*pvfC* mutant was constructed by homologous recombination using the suicide vector pJQ200 [43]. Upstream and downstream flanking regions of pvfC were amplified by PCR (upstream forward: 5′-[TCTAGATGGGGCACACGACGCATCTG]-3′; upstream reverse: 5′-[AAGCTTGCTTCCTGCAGCACCCCGAT]-3′; downstream forward: 5′-[ACTAGTGGATGAGTGCCCAGCAGCGT]-3′; downstream reverse: 5′-[GGATCCGAGCGAGGAAGTCCACCGGC]-3′) and cloned into pJQ200 using XbaI/HindIII/SpeI/BamHI. The recombinant plasmid was transferred to *Pseudomonas* sp. MUP55 by conjugation via *E. coli* ST18 [44]. Transconjugants were selected on LB + gentamicin (30 µg/mL) and confirmed by PCR.

### 4.7. Inhibition of Phytopathogens In Vitro

*Pseudomonas* sp. MUP55 and mutant strains were cultured in LB for 48 h to maximize the concentration of secreted secondary metabolites, consistent with reports that antimicrobial secondary metabolite production in *Pseudomonas* spp. peaks during the transition from late-exponential to stationary growth phase [45,46] (10,000× *g*, 5 min) and supernatants filtered–sterilized (0.22 µm). *E. coli* and *X. campestris* were inoculated in LB with sterile supernatant (1:1 *v*/*v*) and OD_600_ measured over 24 h. For antifungal assays on PDA, bacteria were streaked 2 cm from center; 1 h later, a 5 mm fungal plug was placed at center. Inhibition was assessed after 6 days (*F. oxysporum*) and 4 days (*R. solani*). PIRG (percentage inhibition of radial growth, %) = [1 − (fungal growth near bacteria/fungal growth on opposite side)] × 100%. Cell-free supernatants from 48 h cultures of WT *Pseudomonas* sp. MUP55, M7, and WT Δ*pvfC* were prepared via centrifugation (10,000× *g*, 5 min) and filter sterilization (0.22 µm). *E. coli* and *X. campestris* cells in the exponential phase were inoculated in either supernatant: fresh LB at 1:1 *v*/*v* or in LB only. OD_600_ nm was monitored at 10 min intervals for 24 h in a Varioskan LUX Multimode Microplate Reader (ThermoFisher) at 28 °C, with continuous shaking. Two sterile LB blank wells were monitored throughout the run and remained below OD_600_ nm 0.09, and the mean blank trajectory was subtracted from all wells per timepoint. Total growth per replicate was summarized as the trapezoidal area under the blank-subtracted OD_600_ nm curve (0–24 h). Within each target, the six pairwise supernatant comparisons (LB vs. WT, LB vs. M7, LB vs. ΔpvfC, WT vs. M7, WT vs. ΔpvfC, and M7 vs. ΔpvfC) were tested by Welch’s two-sample *t*-test and Holm-corrected to control the family-wise error rate.

### 4.8. Metabolomic Analysis

Cyclic lipopeptides were extracted from 48 h culture supernatants using ethyl acetate (1:1.1 *v*/*v*, 2 h). Reversed-phase LC used a Waters Acquity I-class UPLC system with HSS-T3 column (1.8 µm, 2.1 × 100 mm). Mobile phase: A (99.9% H_2_O + 0.1% formic acid) and B (99.9% MeCN + 0.1% formic acid); flow rate, 0.25 mL/min; gradient from 100% A to 95% B over 30 min; column temperature, 40 °C. MS was acquired on a Bruker Impact II QToF (ESI positive mode; full scan 80–2000 *m*/*z*; Auto MS/MS; capillary 4.5 kV; drying gas 12.0 L/min; drying temperature 250 °C). Classical molecular networking was performed via GNPS (http://gnps.ucsd.edu) with min pairs cosine 0.7, topK 10, and minimum matched fragments 20. BGC identification used antiSMASH [29,47]. Untargeted comparative metabolomic profiling of this cyclic-lipopeptide-enriched extract was performed on three biological replicates per strain using XCMS with centWave peak detection, obiwarp retention time correction and Welch’s *t*-tests, applying a fold-change threshold of 2 and an exploratory significance threshold of *p* < 0.1, appropriate to untargeted profiling. Features with maximum ion intensity <5000 were filtered out. Given the exploratory, hypothesis-generating purpose of this screen, correction for multiple testing (e.g., Benjamini–Hochberg FDR) was not applied to the resulting feature list; the 1413 features identified (Section 2.6) represent candidates for downstream targeted follow-up, as was subsequently performed for Massetolide A/D via GNPS molecular networking and antiSMASH BGC annotation, rather than confirmed differentially abundant metabolites.

### 4.9. RNA Extraction and Transcriptomic Analysis

WT and Δ*pvfC* strains were grown in parallel in LB at 28 °C, with shaking at 200 rpm, for 24 h to stationary phase, when *Pseudomonas* secondary metabolite biosynthetic and Gac/Rsm-regulated programs are most active [20]. Cultures were then normalized to OD_600_ = 1.0 to standardize cell input across samples and harvested for RNA extraction. Total RNA was extracted from frozen lysates using the Qiagen RNeasy kit according to the manufacturer’s protocol. RNA quality was assessed by Agilent RNA TapeStation (all samples RIN > 6.5). DNase treatment and ribosomal RNA depletion were performed prior to cDNA conversion using an adapted Smart-seq assay with SuperScript™ IV Reverse Transcriptase (ThermoFisher). Full-length cDNAs were amplified using KAPA HiFi DNA Polymerase (Roche Biosystems, Roche, Sydney, NSW, Australia). Libraries were prepared using NEBNext^®^ Ultra™ II FS DNA Library Prep Kit (New England Biolabs), and four biological replicates of each strain were sequenced on Illumina NovaSeq 6000 (150 bp PE) (Illumina, Melbourne, VIC, Australia), yielding an average of 32 million PE reads per sample. The two strains showed comparable growth in LB and were harvested at matched optical density and growth phase.

Read processing and quantification were performed with the nf-core RNA-seq pipeline (version 3.7) within Nextflow (version 22.04.0) [48], using the default star_salmon route: adapter and quality trimming with Trim Galore, v2.2.0, alignment to the *Pseudomonas* sp. MUP55 reference genome (GenBank CP138214) with STAR [49], and transcript-level quantification with Salmon [50]. Gene-level length-scaled counts were generated via tximport. Differential expression analysis was carried out in R v4.4.2 using DESeq2 v1.46.0 (Bioconductor 3.20 [51]), which applies the median-of-ratios method for between-sample normalization and fits a negative binomial generalized linear model to the count data [52]. Significance was assessed using the Wald test, with *p*-values adjusted for multiple testing by the Benjamini–Hochberg procedure. Genes with an adjusted *p*-value < 0.05 and |log_2_ fold change| ≥ 1 were considered differentially expressed.

For the functional category analysis, genes were assigned to one of five high-level functional Superclasses (Metabolism, Energy, RNA processing, Membrane transport, Protein processing) using the BV-BRC functional annotation framework [53]. Genes assigned to multiple Superclasses were included in all relevant categories. Within-category differences in expression between WT and Δ*pvfC* were tested using paired Wilcoxon signed-rank tests (*p* < 0.05); where the assumptions of the paired Wilcoxon test could not be met, paired *t*-tests were used.

### 4.10. β-Galactosidase Reporter Assays

Promoter regions of rsmY and rsmZ were transcriptionally fused to lacZ in reporter plasmids and introduced into *Pseudomonas* sp. MUP55 WT and the ΔpvfC mutant, both of which lack endogenous β-galactosidase activity [54]. β-Galactosidase activity was quantified by a one-step assay [55]: overnight cultures were diluted and grown to OD_600_ = 1.0. A 500 µL volume was lysed by adding 10 µL of 0.1% SDS and 10 µL of chloroform (vortex 15 s). The mixture was incubated at 37 °C after adding 100 µL of 4 mg/mL ONPG (o-nitrophenyl-β-d-galactopyranoside; Sigma-Aldrich, Bayswater, VIC, Australia). OD_420_ nm was measured. Activity (Miller units) = (1000 × OD420)/T/OD600; T = reaction time (min).

### 4.11. Statistical Analysis

All experiments were performed with at least three biological replicates unless otherwise stated, and data are presented as mean ± standard deviation (SD). Growth-inhibition and bacteriostatic assays were compared on the area under the OD_600_ nm curve using Welch’s *t*-test with Holm correction for multiple comparisons. Transcriptomic functional-category comparisons used paired Wilcoxon signed-rank tests. Differential gene expression was assessed in DESeq2 using the Wald test with Benjamini–Hochberg correction (adjusted *p* < 0.05, |log_2_ fold-change| ≥ 1), and comparative metabolomics used Welch’s *t*-tests with a fold-change threshold of 2 (exploratory *p* < 0.1) [56]. Unless otherwise indicated, *p* < 0.05 was considered statistically significant. All analyses were performed in R (version 4.4.2).

## 5. Conclusions

This study provides a comprehensive polyphasic characterization of *Pseudomonas* sp. MUP55, isolated from rainwater in Western Australia. Whole-genome-based taxonomic analyses, including dDDH, ANI, and phenotypic and chemotaxonomic profiling, support classification of *Pseudomonas* sp. MUP55 as a distinct species within the *P. fluorescens* group. *Pseudomonas* sp. MUP55 produces Massetolide A/D as a leading candidate antimicrobial compound, contributing to both antibacterial and antifungal biocontrol activity, although the two isomers could not be conclusively distinguished by the available mass spectrometry data, and their individual contributions remain to be confirmed with purified standards. Transcriptomic and metabolomic analyses of a *ΔpvfC* mutant revealed that the *pvf* gene cluster contributes to both the regulation of specialized metabolism and to secreted growth-inhibitory activity, including an uncoupling of the small RNAs *rsmY* and *rsmZ* within the Gac/Rsm regulatory cascade. Several individual regulatory genes examined in this network did not reach statistical significance after correction for multiple testing, and these findings should be interpreted as hypotheses for future validation rather than established mechanisms. Collectively, these results position *Pseudomonas* sp. MUP55 as a taxonomically novel and mechanistically well-characterized candidate biocontrol agent, while highlighting specific directions for future work, including genetic complementation of the GacA and *pvfC* mutants, direct testing of purified Massetolide A/D, and transcript-level validation of the proposed pvf–Gac/Rsm regulatory link.

## Figures and Tables

**Table 1:** Differential gene expression between WT and Δ*pvfC* strains for the siderophore pathways. Log_2_fold changes are expressed as Δ*pvfC*/WT.

**The NRPS-Independent Siderophore Pathway**				
**Locus Tag**	**Gene**	**Function**	**log_2_Fold(ΔpvfC/WT)**	***p*** **adj**
SC318_RS18895	*trbA*	IucA/IucC family protein	10.97075	1.28 × 10^−11^
SC318_RS18905	*trbC*	TonB-dependent receptor	11.15405	0.053 ^†^
**The NRPS-Dependent Siderophore Pathway**				
SC318_RS10910	-	Non-ribosomal peptide synthase/polyketide synthase	−8.12384	8.21× 10^−5^
SC318_RS10920	*fpvA*	TonB-dependent siderophore receptor	−6.6313	2.42 × 10^−4^
SC318_RS10945	-	Pyoverdine-tailoring dipeptidase-like protein PvdM	−5.22578	NA ^‡^
SC318_RS10950	-	PvdJ/PvdD/PvdP-like protein	1.681854	3.35 × 10^−3^
SC318_RS10955	-	Fic family protein	−8.72194	3.23 × 10^−6^

^†^ Borderline—just above the *p*adj < 0.05 threshold; interpret with appropriate caution. ^‡^ Excluded by DESeq2 independent filtering (low mean count); *p*adj not calculated.

**Table 2:** Differential gene expression between WT and Δ*pvfC* strains for genes involved in Massetolide biosynthesis and regulation.

Locus Tag	Gene	Function	log_2_Fold(ΔpvfC/WT)	*p*adj
SC318_RS15655	*gacS*	Signal transduction histidine-protein kinase	1.38	0.456
SC318_RS09875	*gacA*	GacA response regulator	0.65	0.914
SC318_RS16815	*luxR1*	Transcriptional regulator	1.68	0.614
SC318_RS15380	*luxR2*	Transcriptional regulator	8.97	0.120
SC318_RS16810	massA	Non-ribosomal peptide synthetase	6.06	NA ^‡^
SC318_RS15400	massB	Non-ribosomal peptide synthetase	−2.38	0.042 *
SC318_RS15395	massC	Non-ribosomal peptide synthetase	2.72	NA ^‡^
SC318_RS16820	*pleC*	Efflux transporter outer membrane subunit	−1.69	0.264
SC318_RS16275	*prtR*	Transmembrane regulatory gene (anti-sigma factor)	−10.69	0.064

* Significant at *p*adj < 0.05. ^‡^ Excluded by DESeq2 independent filtering (low mean count); *p*adj not calculated.

## Data Availability

The genome sequence of *Pseudomonas* sp. MUP55 was deposited in GenBank under accession CP138214, with raw reads under SRA accession SRR26637682. RNA-seq data were deposited in the NCBI Sequence Read Archive under BioProject accession PRJNA1497945 (BioSample accessions SAMN61825623–SAMN61825630); per NCBI guidance, this BioProject accession should be cited in place of individual run accessions for improved searchability in Entrez.

## Supplementary Materials

The following supporting information can be downloaded at: https://www.mdpi.com/article/10.3390/ijms27156749/s1.
